# High prevalence of low back pain among young basketball players with lower extremity pain: a cross-sectional study

**DOI:** 10.1186/s13102-020-00189-6

**Published:** 2020-07-06

**Authors:** Yutaka Yabe, Yoshihiro Hagiwara, Takuya Sekiguchi, Haruki Momma, Masahiro Tsuchiya, Kenji Kanazawa, Nobuyuki Itaya, Shinichirou Yoshida, Yasuhito Sogi, Toshihisa Yano, Takahiro Onoki, Eiji Itoi, Ryoichi Nagatomi

**Affiliations:** 1 Department of Orthopaedic Surgery, Tohoku University School of Medicine, 1-1 Seiryo-machi, Aoba-ku, Sendai, 980-8574 Japan; 2 Department of Medicine and Science in Sports and Exercise, Tohoku University School of Medicine, 2-1 Seiryo-machi, Aoba-ku, Sendai, 980-8575 Japan; 3 Department of Nursing, Faculty of Health Science, Tohoku Fukushi University, 1-8-1 Kunimi, Aoba-ku, Sendai, 981-8522 Japan; 4 Division of Biomedical Engineering for Health and Welfare, Tohoku University Graduate School of Biomedical Engineering, 2-1 Seiryo-machi, Aoba-ku, Sendai, 980-8575 Japan

**Keywords:** Ankle pain, Basketball, Knee pain, Low back pain, Lower extremity pain, School-aged

## Abstract

**Background:**

Low back pain (LBP) is a common problem among young basketball players in addition to lower extremity injuries. However, studies that focus on LBP with lower extremity pain are limited. From the perspective of the kinematic chain, disrupted lower extremity function can lead to LBP. The association between these two symptoms in basketball players, however, has not been reported. Therefore, this study aimed to clarify the association between lower extremity pain and LBP among young basketball players.

**Methods:**

This cross-sectional study was conducted on school-aged basketball players (*n* = 592). Information regarding their sporting activities was collected using a self-reported questionnaire. Musculoskeletal pain such as low back, knee, and ankle pain was assessed. The sports players with knee and/or ankle pain were defined as having lower extremity pain. Multivariate logistic regression analysis was used to assess the association between lower extremity pain and LBP. Odds ratios (OR) and 95% confidence intervals (95% CI) were calculated. The associations of knee or ankle pain with LBP were similarly assessed.

**Results:**

School-aged basketball players had a point prevalence of 12.8% for LBP. Compared with the players without lower extremity pain, the players with lower extremity pain had higher rates of LBP, with an adjusted OR (95% CI) of 6.21 (3.57–10.80). There was also a significant association of knee and ankle pain with LBP. Compared with the players without knee or ankle pain, the adjusted ORs (95% CI) for LBP were 4.25 (2.55–7.07) in the players with knee pain and 3.79 (2.26–6.36) in the players with ankle pain.

**Conclusions:**

Lower extremity pain was associated with LBP among school-aged basketball players. Further research is needed to clarify the mechanism of this association, which will provide useful information for prevention and treatment of LBP among young basketball players.

## Background

Low back pain (LBP) is a common problem among young sports players [1]. LBP negatively impacts athletic performance, and sometimes interferes with young sports players’ continued participation in a sport [2]. Therefore, clarifying the factors related to LBP is necessary for the effective management of this problem in young sports players. Because different sports require specific postures that place a load on the low back, some sports have a higher prevalence of LBP [3].

Basketball is a major global sport with approximately 450 million players [4]. Basketball includes intensive movements, which can often lead to several injuries [5]. For example, injuries of the lower extremities, particularly the knees and ankles, are the most common injuries among basketball players [6]. Although little attention has been paid to LBP among basketball players; some authors report that basketball players have a high prevalence of LBP [3, 7]. Basketball involves a high frequency of jumps and landings with ball-handling [5, 8]. Furthermore, basketball includes rotational and asymmetrical movements [4]. These motions are considered to lead to high LBP rates among basketball players [7]. Although reports that have investigated factors related to LBP among basketball players are rare, generally, age [9], sex [8], body mass index (BMI) [7], and intensity/frequency of training [8] are thought to be related to LBP.

Recently, the kinematic chain has received increased attention from athletes because a disturbance in the kinematic chain is thought to cause sports injuries [10]. Furthermore, some reports have demonstrated correlations with other sports between pain in various body parts, such as low back and elbow pain, as well as neck and shoulder pain [10, 11], which are thought to arise from disrupted kinematic chains [12]. In addition, athletes with ligamentous laxity due to previous trauma of the lower extremities were reported to have a higher rate of LBP [12]. Forces transfer from the lower extremities through the trunk to the upper extremities during body motions [13] and thus we hypothesised that loss of function in the lower extremities due to pain may lead to LBP among basketball players. To prevent and treat LBP in basketball players, it may be useful to elucidate the associations of lower extremity pain with LBP. Nevertheless, there have been no reports evaluating these associations among basketball players. The present study therefore aimed to assess the association of lower extremity pain, such as knee and ankle pain, with LBP among school-aged basketball players.

## Methods

### Participants

This study forms one part of the comprehensive cross-sectional study of young sports players (age, 6–15 years) belonging to teams registered in the amateur sports association in Miyagi prefecture, Japan [14]. The association includes a variety of sports teams and has registered 25,469 sports players. Information that related to sporting activity participation was assessed with a self-reported questionnaire (Additional file 1), which was mailed to all sports players along with an informed consent form in October 2014. Although participants were instructed to answer the questionnaires by themselves, parents were allowed to assist the younger participants.

### Low back and lower extremity pain

The outcome of interest was LBP and the main predictor was lower extremity pain. Low back, knee, and ankle pain were evaluated by the self-reported questionnaire [14]. The question was “Do you have pain in any parts of your body now? If yes, please mark the parts where you have pain with a circle (multiple answers were allowed).” The body parts were illustrated with a diagram that included names. The participants with knee and/or ankle pain were defined as having lower extremity pain [15]..

### Covariates

The following variables were assessed by the self-reported questionnaire and were included in the analysis as covariates because they were considered potential cofounding factors: sex (boy or girl), age, BMI: calculated using self-reported height and weight), team level (recreation, local competition, prefectural competition, Tohoku district competition, national competition), practice days per week, practice hours per day on weekdays and on weekends, participation frequency in games (never, seldom, sometimes, and often), and practice intensity (low-to-medium, high) [16].

### Statistical analysis

Continuous variables were presented as medians with interquartile range (IQR) and categorical variables were presented as proportions and percentages (%). The prevalence of low back and lower extremity pain was also presented as proportions and percentages (%). Univariate and multivariate logistic regression analyses were examined to assess the associations of lower extremity pain with LBP, and odds ratios (ORs) and 95% confidence intervals (95% CIs) were calculated [17]. Furthermore, the association of knee or ankle pain with LBP was assessed in the same manner. SPSS 24.0 (SPSS Japan Inc., Tokyo, Japan) was used for the statistical analysis and values of *P* <  0.05 were considered statistically significant.

## Results

**Fig. 1:**
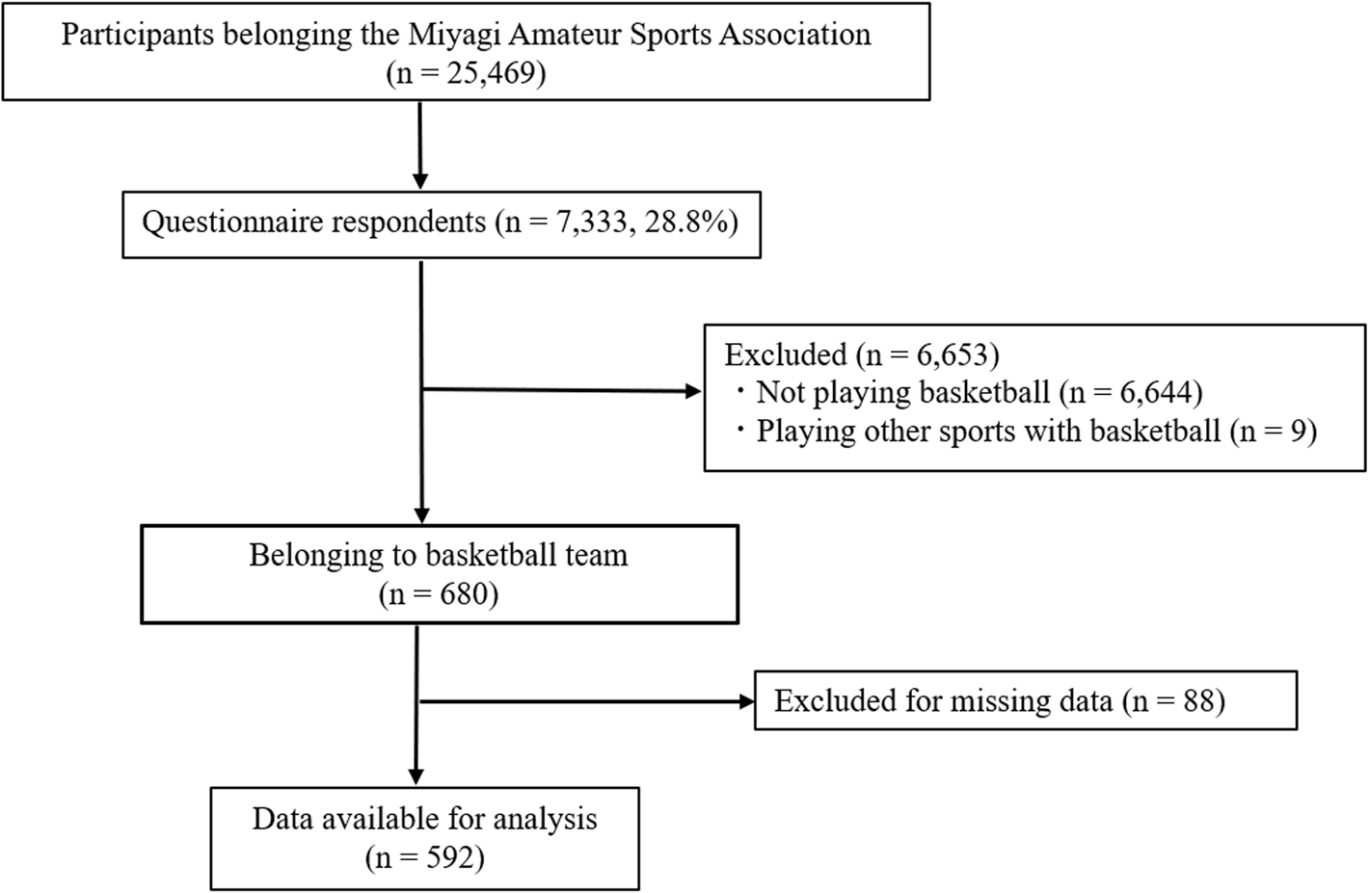
Flow chart of the study design

Among the 25,469 registered sports players, 7333 (28.8%) replied to the questionnaire with consent to participate in this study before the end of December 2014. Of them, 680 sports players belonged to basketball teams. Respondents who had missing data were excluded (*n* = 88), and a final sample size of 592 young basketball players was included (Fig. 1). The participants’ baseline characteristics are shown in Table 1. The participants’ median age was 13 (IQR: 12–14) years. The point prevalence of LBP, knee pain, and ankle pain among school-aged basketball players was 12.8% (76/592), 25.2% (149/592), and 21.5% (127/592), respectively. Defined lower extremity pain was found in 36.7% (217/592) of the participants. There was a significant association between lower extremity pain and LBP. Compared with the participants without lower extremity pain, those with lower extremity pain had higher rates of LBP, and the adjusted OR (95% CI) was 6.21 (3.57–10.80). There was also a significant association between knee/ankle pain and LBP. Compared with the participants without knee/ankle pain, the adjusted ORs (95% CI) for LBP were 4.25 (2.55–7.07) for the participants with knee pain and 3.79 (2.26–6.36) in the participants with ankle pain (Table 2).

**Table 1:** Baseline characteristics of the participants

Variables	Categories	Median (IQR)	Total, N (%)	Low back pain, N (%)
Total			592	76
Gender	Male		332 (56.1)	35 (46.1)
	Female		260 (43.9)	41 (53.9)
Age (years)		13.0 (12.0, 14.0)		
Body mass index		18.3 (16.7, 19.8)		
Team levels	Recreation		30 (5.1)	2 (2.6)
	Local competition		376 (63.5)	49 (64.5)
	Prefectural competition		169 (28.5)	22 (28.9)
	Tohoku district or national competition		17 (2.9)	3 (3.9)
Training per week (days)		5.0 (3.5, 6.0)		
Practice per day weekdays (hours)		2.0 (2.0, 2.5)		
Practice per day weekends (hours)		3.0 (3.0, 4.0)		
Frequency of participation in games	never		31 (5.2)	3 (3.9)
	seldom		57 (9.6)	7 (9.2)
	sometimes		175 (29.6)	21 (27.6)
	often		329 (55.6)	45 (59.2)
Practice intensity	Low-to-medium		203 (34.3)	18 (23.7)
	High		389 (65.7)	58 (76.3)

**Table 2:** Association of lower extremity pain with low back pain among young basketball players

	Lower extremity pain		
	Absence (*n* = 375)	Presence (*n* = 217)	*p* value
Low back pain, n (%)	20 (5.3)	56 (25.8)	
Crude ORs (95% CI)	1	6.17 (3.59–10.63)	< 0.001
Adjusted ORs (95% CI)	1	6.26 (3.60–10.89)	< 0.001
	Knee pain		
	Absence (*n* = 443)	Presence (*n* = 149)	*p* value
Low back pain, n (%)	36 (8.1)	40 (26.8)	
Crude ORs (95% CI)	1	4.15 (2.52–6.82)	< 0.001
Adjusted ORs (95% CI)	1	4.26 (2.55–7.10)	< 0.001
	Ankle pain		
	Absence (*n* = 465)	Presence (*n* = 127)	*p* value
Low back pain, n (%)	41 (8.8)	35 (27.6)	
Crude ORs (95% CI)	1	3.93 (2.38–6.51)	< 0.001
Adjusted ORs (95% CI)	1	3.92 (2.33–6.61)	< 0.001

Adjusted for sex, age, body mass index, team levels, number of days for training per week, number of hours in practice per day on weekdays and weekends, frequency of participation in games, and practice intensity

*OR* odds ratio, *CI* confidence interval

## Discussion

Our main finding was that lower extremity pain was associated with LBP in young basketball players. Further, both knee and ankle pain were associated with LBP among the young basketball players.

Although reports of LBP among young basketball players are rare, one study showed a 37.9% lifetime prevalence of LBP among school-aged basketball players [18]. Another report showed a lifetime prevalence of 45.4% and a one-week prevalence of 19.8% among young basketball players with a mean age of 14.9 years [5]. Further, our study showed that the point prevalence of LBP among school-aged basketball players was 12.8%. Because basketball players are considered to have a high prevalence of LBP, even young players, elucidating the related factors, and identifying effective prevention and treatment methods for LBP among young basketball players is necessary.

Although there are only a few reports, the association between lower extremity pain and LBP or back pain has been demonstrated in other sports, such as baseball and football [15, 17]. The lower extremities provide a stable base for lumbar movement [19] and control the ground reaction forces to the lower back [12]. Lower extremity pain can interrupt these processes and may transfer excessive loads to the lumbar spine, which can lead to LBP. Overall, 36.4% of players had lower extremity pain in our study. Players are required to repeat sudden stops, turns, jumps, and landings in basketball [5] and thus have a high frequency of lower extremity injuries [20]. Further, 25.8% of players with lower extremity pain had LBP, which was significantly higher compared with 5.3% of players without lower extremity pain. Longitudinal studies should be conducted to clarify the association of preceding lower extremity pain with LBP, which may provide useful information to clinicians and coaches to treat and prevent LBP among young basketball players.

Considering the types of lower extremity pain, some authors have reported that the knee is the most commonly injured body part in basketball players [20, 21]. During basketball movements, players often keep their knees bent [4] and knee pain can disrupt this function. Insufficient knee flexion upon landing reduces shock absorption and increases vertical forces to the low back [22]. In our study, 25.2% of young basketball players reported knee pain. Among those, 26.8% had LBP, which was significantly higher compared with 8.1% among players without knee pain. This result suggests that knee pain increases the load on the low back and leads to LBP in young basketball players. Suter et al. (2001) showed the association between diminished knee extensor strength and LBP in their cross-sectional study [23]. Knee pain reduces knee extension strength and disrupts knee function, which results in an increased load on the low back. These hypotheses should be assessed in future studies, which may provide an effective approach to treat and prevent LBP in young basketball players.

Ankle injuries are also common among basketball players [6]. In basketball, ankle sprains are major injuries that are often caused by jumps or landings, because it is common to play in areas with high player concentrations [20]. Ankle sprains also have high re-injury rates among basketball players [21] and can lead to a high prevalence of ankle pain. Brantingham et al. (2006) reported that decreased range of motion of the ankle was associated with LBP, which was due to the loss of shock absorption at the ankle [24]. In our study, the prevalence of ankle pain was 21.5%. Among those with ankle pain, 27.6% had LBP, which was significantly higher compared with 8.8% of players without ankle pain. Preceding ankle pain can be associated with the onset of LBP, which should be assessed in future longitudinal studies. Although only a few studies have reported on the associations of ankle function with LBP [25], the results of our study provide an insight into the association of disrupted ankle function with LBP onset. Further research to investigate this association can aid in the development of a strategy for the treatment and prevention of LBP among young basketball players.

This study has several limitations. First, we distributed the questionnaires by mail. As a result, the response rate was not high. Furthermore, the sample size was not calculated at the start of this study because the number of respondents could not be predetermined. Second, low back and lower extremity pain were assessed using a self-reported questionnaire, but the intensity and duration of pain were not assessed. Additionally, the reliability and validity of this questionnaire were not evaluated in this study. Questionnaires specific to low back and lower extremity pain should be considered in future studies. Finally, this study employed a cross-sectional design and there remained the possibility of reverse causality.

## Conclusion

Elementary and middle school-aged basketball players with lower extremity pain have a high prevalence of LBP. Further research is needed to clarify the mechanism of this association, which will give useful information for prevention and treatment of LBP among young basketball players.

## Supplementary information

**Additional file 1.**

## Data Availability

The datasets used and/or analysed during the current study are available from the corresponding author on reasonable request.

## Abbreviations

BMI: Body mass index
CI: Confidence interval
LBP: Low back pain
OR: Odds ratio
