# Supplementary material for: High prevalence of low back pain among young basketball players with lower extremity pain: a cross-sectional study
Source: BMC Sports Sci Med Rehabil. 2020 Jul 6;12:40. doi: 10.1186/s13102-020-00189-6 (PMC7339456; doi:10.1186/s13102-020-00189-6)

Questionnaires

1. How old are you? ________ years old
2. Please tell me your gender. (boy ・girl)
3. Please tell me your height and weight.

   Height ________ cm, Wight ________ kg

4. Please tell me your age when you started the present sports.

   ________ years old

5. How often do you participate in games?

   (never, seldom, sometimes, often)

6. Please tell me sports discipline you participate in. If there are no choices, please fill in the parenthesis.

   a. baseball, b. football, c. volleyball, d. mini-basketball, e. basketball, f. judo, g. kendo, h. karate, i. softball, j. handball, k. tennis, l. track and field, m. ski, n. table tennis, o. badminton, p others (________)

7. Please tell me your team levels.

   (recreation, local competition, prefectural competition, Tohoku district competition, national competition)

8. How many days do you have practice per week?

______________ days/week

9. How many hours do you have practice per day on weekdays or weekends?

Weekdays: _____________ hours

Weekends: _____________ hours

10. How do you feel the practice?

(not hard, hard)

11. Do you have pain in any pats of your body? If yes, please mark the parts where you have pain with a circle (multiple answers were allowed).

(head, face, rt shoulder, lt shoulder, rt elbow, lot elbow, rt hand, lt hand, rt hip, lt hip, rt knee, lt knee, rt ankle, lt ankle, back, low back, rt buttock, lt buttock)

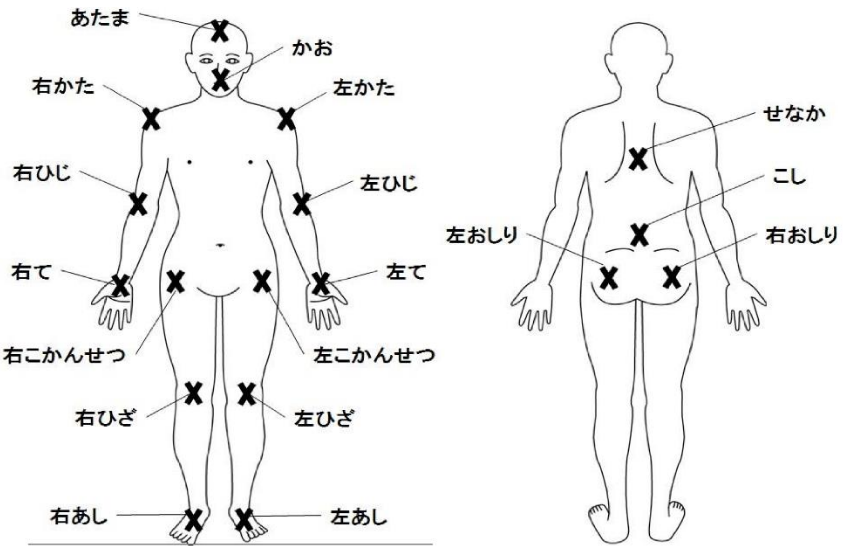

Supplement: Supplementary file 1 — Additional file 1. [file 13102_2020_189_MOESM1_ESM.pdf]
